# Supplementary material for: Advancements in Crop PUFAs Biosynthesis and Genetic Engineering: A Systematic and Mixed Review System
Source: Int J Mol Sci. 2025 Apr 8;26(8):3462. doi: 10.3390/ijms26083462 (PMC12027219; doi:10.3390/ijms26083462)
Supplement: Supplementary file 1 [file ijms-26-03462-s001.zip › ijms-3469814-supplementary.pdf]

Table S1. The list of 72 most recent and related research articles on crop essential oil biosynthesis and functional food during, 2013-2024

S/n	Reference	Total citation count	Adjusted citation count
1	*Schübel, R., Jaudszus, A., Krüger, R., Roth, A., Klempt, M., & Barth, S. W. (2017). Dietary essential α-linolenic acid and linoleic acid differentially modulate TNFα-induced NFκB activity in FADS2-deficient HEK-293 cells/ 68(5), 553-559.*	9	1.3
2	*Burns-Whitmore, B., Froyen, E., Heskey, C., Parker, T., & San Pablo, G. (2019). Alpha-linolenic and linoleic fatty acids in the vegan diet: do they require dietary reference intake/adequate intake special consideration?.Nutrients, 11(10), 2365.*	151	30.2
3	*Pottel, L., Lycke, M., Boterberg, T., Foubert, I., Pottel, H., Duprez, F.,& Debruyne, P. R. (2014). Omega-3 fatty acids: physiology, biological sources and potential applications in supportive cancer care. Phytochemistry reviews, 13, 223-244.*	46	4.6
4	*Hooper, L., Abdelhamid, A., Brainard, J., Deane, K. H., & Song, F. (2019). Creation of a database to assess effects of omega-3, omega-6 and total polyunsaturated fats on health: methodology for a set of systematic reviews. BMJ open, 9(5), e029554.*	12	2.4
5	*EFSA Panel on Dietetic Products, Nutrition and Allergies (NDA). (2015). Scientific Opinion on the safety of refined Buglossoides oil as a novel food ingredient. EFSA Journal, 13(2), 4029.*	3	0.3
6	*Britten-Jones, A. C., Craig, J. P., & Downie, L. E. (2023). Omega-3 polyunsaturated fatty acids and corneal nerve health: Current evidence and future directions. The Ocular Surface, 27, 1-12.*	12	12
7	*Konda, A. R., Nazarenus, T. J., Nguyen, H., Yang, J., Gelli, M., Swenson, S.,& Cahoon, E. B. (2020). Metabolic engineering of soybean seeds for enhanced vitamin E tocochromanol content and effects on oil antioxidant properties in polyunsaturated fatty acid-rich germplasm. Metabolic Engineering, 57, 63-73.*	44	11
8	*Ursin, V., Froman, B., Nava, A. J., & Gonzales, J. (2013). U.S. Patent No. 8,378,186. Washington, DC: U.S. Patent and Trademark Office.*	60	5.5
9	*Lee, J. M., Lee, H., Kang, S., & Park, W. J. (2016). Fatty acid desaturases, polyunsaturated fatty acid regulation, and biotechnological advances. Nutrients, 8(1), 23.*	424	53
10	*Djuricic, I., & Calder, P. C. (2021). Beneficial outcomes of omega-6 and omega-3 polyunsaturated fatty acids on human health: An update for 2021. Nutrients, 13(7), 2421.*	562	187.3
11	*Neufingerl, N., & Eilander, A. (2021). Nutrient intake and status in adults consuming plant-based diets compared to meat-eaters: a systematic review. Nutrients, 14(1), 29.*	252	84
12	*Sherratt, S. C., Libby, P., Budoff, M. J., Bhatt, D. L., & Mason, R. P. (2023). Role of omega-3 fatty acids in cardiovascular disease: the debate continues. Current atherosclerosis reports, 25(1), 1-17.*	59	59
13	*Saini, R. K., & Keum, Y. S. (2018). Omega-3 and omega-6 polyunsaturated fatty acids: Dietary sources, metabolism, and significance—A review. Life sciences, 203, 255-267.*	1157	192.8
14	*Backes, J., Anzalone, D., Hilleman, D., & Catini, J. (2016). The clinical relevance of omega-3 fatty acids in the management of hypertriglyceridemia. Lipids in Health and Disease, 15, 1-12.*	218	27.3
15	*Bojková, B., Winklewski, P. J., & Wszedybyl-Winklewska, M. (2020). Dietary fat and cancer—which is good, which is bad, and the body of evidence. International journal of molecular sciences, 21(11), 4114.*	133	33.3
16	*Ma, Z., & He, W. (2023). Fatty acids and pregnancy-induced hypertension: a Mendelian randomization study. Lipids in Health and Disease, 22(1), 131.*	4	4

17	*Fan, R., Li, L., Cai, G., Ye, J., Liu, M., Wang, S., & Li, Z. (2019). Molecular cloning and function analysis of FAD2 gene in Idesia polycarpa. Phytochemistry, 168, 112114.*	17	3.4
18	*Liu, G., Wu, Z., Shang, X., Peng, Y., & Gao, L. (2022). Overexpression of PvFAD3 Gene from Plukenetia volubilis Promotes the Biosynthesis of α-Linolenic Acid in Transgenic Tobacco Seeds. Genes, 13(3), 450.*	6	3
19	*Hu, X. D., Pan, B. Z., Fu, Q., Niu, L., Chen, M. S., & Xu, Z. F. (2018). De novo transcriptome assembly of the eight major organs of Sacha Inchi (Plukenetia volubilis) and the identification of genes involved in α-linolenic acid metabolism. BMC genomics, 19, 1-14.*	19	3.2
20	*Park, H., Graef, G., Xu, Y., Tenopir, P., & Clemente, T. E. (2014). Stacking of a stearoyl-ACP thioesterase with a dual-silenced palmitoyl-ACP thioesterase andΔ 12 fatty acid desaturase in transgenic soybean. Plant biotechnology journal, 12(8), 1035-1043.*	17	1.7
21	*Grimberg, Å., Carlsson, A. S., Marttila, S., Bhalerao, R., & Hofvander, P. (2015). Transcriptional transitions in Nicotiana benthamiana leaves upon induction of oil synthesis by WRINKLED1 homologs from diverse species and tissues. BMC plant biology, 15, 1-17.*	120	13.3
22	*Burdziej, A., Pączkowski, C., Destrac-Irvine, A., Richard, T., Cluzet, S., & Szakiel, A. (2019). Triterpenoid profiles of the leaves of wild and domesticated grapevines. Phytochemistry Letters, 30, 302-308.*	9	1.8
23	*Knothe, G., Razon, L. F., & de Castro, M. E. G. (2019). Fatty acids, triterpenes and cycloalkanes in ficus seed oils. Plant Physiology and Biochemistry, 135, 127-131.*	11	2.2
24	*Kodahl, N., & Sørensen, M. (2021). Sacha inchi (Plukenetia volubilis L.) is an underutilized crop with a great potential. Agronomy, 11(6), 1066.*	60	20
25	*Furse, S., Martel, C., Yusuf, A., Shearman, G. C., Koch, H., & Stevenson, P. C. (2023). Sterol composition in plants is specific to pollen, leaf, pollination and pollinator. Phytochemistry, 214, 113800.*	4	4
26	*Guan, H. Y., Su, P., Zhao, Y. J., Zhang, X. N., Dai, Z. B., Guo, J., ... & Huang, L. Q. (2018). Cloning and functional analysis of two sterol-C24-methyltransferase 1 (SMT1) genes from Paris polyphylla. Journal of Asian natural products research, 20(7), 595-604.*	12	2
27	*Ayerdi Gotor, A., Berger, M., Labalette, F., Centis, S., Daydé, J., & Calmon, A. (2014). Oleic conversion effect on the tocopherol and phytosterol contents in sunflower oil. Phyton, 83, 319-324.*	10	1
28	*Castellanos-Jankiewicz, A., del Bosque-Plata, L., & Tejero, M. E. (2014). Combined effect of plant sterols and dietary fiber for the treatment of hypercholesterolemia. Plant foods for human nutrition, 69, 93-100.*	28	2.8
29	*Liu, L., Wang, D., Hua, J., Kong, X., Wang, X., Wang, J.,& Chen, Z. (2023). Genetic and Morpho-Physiological Differences among Transgenic and No-Transgenic Cotton Cultivars. Plants, 12(19), 3437.*	3	3
30	*Yin, Y., Gao, L., Zhang, X., & Gao, W. (2018). A cytochrome P450 monooxygenase responsible for the C-22 hydroxylation step in the Paris polyphylla steroidal saponin biosynthesis pathway. Phytochemistry, 156, 116-123.*	37	6.2
31	*Kamisah, Y., Periyah, V., Lee, K. T., Noor-Izwan, N., Nurul-Hamizah, A., Nurul-Iman, B. S.,& Qodriyah, H. M. S. (2015). Cardioprotective effect of virgin coconut oil in heated palm oil diet-induced hypertensive rats. Pharmaceutical Biology, 53(9), 1243-1249.*	51	5.7
32	*Luisa Hernández, M., Dolores Sicardo, M., Arjona, P. M., & Martínez-Rivas, J. M. (2020). Specialized functions of olive FAD2 gene family members related to fruit development and the abiotic stress response. Plant and Cell Physiology, 61(2), 427-441.*	29	7.3
33	*Panchal, S. K., Carnahan, S., & Brown, L. (2017). Coconut products improve signs of diet-induced metabolic syndrome in rats. Plant Foods for Human Nutrition, 72, 418-424.*	21	3
34	*Liu, B., Chen, K., Chen, X., Wang, J., Shu, G., Ping, Z., & Zhang, S. (2023). Health outcomes associated with phytosterols: An umbrella review of*	2	2

	systematic reviews and meta-analyses of randomized controlled trials. Phytomedicine, 155151.		
35	*Huang, A. H. (2018). Plant lipid droplets and their associated proteins: potential for rapid advances. Plant physiology, 176(3), 1894-1918.*	206	34.3
36	*Scholz, P., Chapman, K. D., Mullen, R. T., & Ischebeck, T. (2022). Finding new friends and revisiting old ones–how plant lipid droplets connect with other subcellular structures. New Phytologist, 236(3), 833-838.*	18	9
37	*Kitaoka, N., Lu, X., Yang, B., & Peters, R. J. (2015). The application of synthetic biology to elucidation of plant mono-, sesqui-, and diterpenoid metabolism. Molecular plant, 8(1), 6-16.*	110	12.2
38	*Liu, F., Wang, P., Xiong, X., Zeng, X., Zhang, X., & Wu, G. (2021). A review of nervonic acid production in plants: prospects for the genetic engineering of high nervonic acid cultivars plants. Frontiers in Plant Science, 12, 626625.*	49	16.3
39	*Bai, Y., Zhu, X., Guo, X., Zhang, W., Zhang, G., Chen, H., & Zhang, Q. (2021). Molecular cloning and functional characterization of GmAAPTs from soybean (Glycine max). Plant Signaling & Behavior, 16(1), 1845048.*	3	1
40	*Sun, M., Zhang, J., Wang, N., Wei, X., Fang, H., Ding, X.,& Wang, X. (2024). Genome-Wide Identification, Phylogenetic and Expression Pattern Analysis of Fatty Acid Desaturase Genes in Castor (Ricinus communis L.). Tropical Plant Biology, 17(1), 52-64.*	1	-
41	*Matzner, M., Launhardt, L., Barth, O., Humbeck, K., Goss, R., & Heilmann, I. (2023). Inter-Organellar Effects of Defective ER-Localized Linolenic Acid Formation on Thylakoid Lipid Composition, Non-Photochemical Quenching of Chlorophyll Fluorescence and Xanthophyll Cycle Activity in the Arabidopsis fad3 Mutant. Plant and Cell Physiology, pcad141.*	3	3
42	*Tran, H. T. D., Le, N. T., Khuat, V. L. U., & Nguyen, T. T. H. (2019). Identification and functional characterization of a soybean (Glycine max) thioesterase that acts on intermediates of fatty acid biosynthesis. Plants, 8(10), 397.*	7	1.4
43	*Ma, W., Kong, Q., Mantyla, J. J., Yang, Y., Ohlrogge, J. B., & Benning, C. (2016). 14-3-3 protein mediates plant seed oil biosynthesis through interaction with AtWRI1. The Plant Journal, 88(2), 228-235.*	70	8.8
44	*Tang, Y., Huang, J., Ji, H., Pan, L., Hu, C., Qiu, X., & Qiao, L. (2022). Identification of AhFatB genes through genome-wide analysis and knockout of AhFatB reduces the content of saturated fatty acids in peanut (Arichis hypogaea L.). Plant Science, 319, 111247.*	14	7
45	*Xue, M., Guo, T., Ren, M., Wang, Z., Tang, K., Zhang, W., & Wang, M. (2019). Constitutive expression of chloroplast glycerol-3-phosphate acyltransferase from Ammopiptanthus mongolicus enhances unsaturation of chloroplast lipids and tolerance to chilling, freezing and oxidative stress in transgenic Arabidopsis. Plant Physiology and Biochemistry, 143, 375-387.*	29	5.8
46	*Xie, D., Dai, Z., Yang, Z., Tang, Q., Deng, C., Xu, Y.,& Su, J. (2019). Combined genome-wide association analysis and transcriptome sequencing to identify candidate genes for flax seed fatty acid metabolism. Plant Science, 286, 98-107.*	43	8.6
47	*Kobayashi, K., Fujii, S., Sato, M., Toyooka, K., & Wada, H. (2015). Specific role of phosphatidylglycerol and functional overlaps with other thylakoid lipids in Arabidopsis chloroplast biogenesis. Plant cell reports, 34, 631-642.*	65	7.2
48	*Liu, F., Ma, L., Wang, Y., Li, Y., Zhang, X., Xue, F.,& Sun, J. (2019). GhFAD2–3 is required for another development in Gossypium hirsutum. BMC plant biology, 19, 1-17.*	22	4.4
49	*Jarvis, B. A., Romsdahl, T. B., McGinn, M. G., Nazarenus, T. J., Cahoon, E. B., Chapman, K. D., & Sedbrook, J. C. (2021). CRISPR/Cas9-induced fad2 and rod1 mutations stacked with fae1 confer high oleic acid seed oil in pennycress (Thlaspi arvense L.). Frontiers in plant science, 12, 652319.*	41	13.7
50	*Langyan, S., Yadava, P., Sharma, S., Gupta, N. C., Bansal, R., Yadav, R.,& Kumar, A. (2022). Food and nutraceutical functions of sesame oil: An underutilized crop for nutritional and health benefits. Food chemistry, 389, 132990.*	59	29.5

51	*Zhao, G., Zhao, J., Zhang, X., Wang, S., Fu, D., & Chen, M. (2022). Yacon (Smallanthus sonchifolius) tuber: A novel and promising feedstock for enhanced high-value docosahexaenoic acid production by Schizochytrium sp. Industrial Crops and Products, 188, 115597.*	9	4.5
52	*Zimmer, B., Angioni, C., Osthues, T., Toewe, A., Thomas, D., Pierre, S. C., & Sisignano, M. (2018). The oxidized linoleic acid metabolite 12, 13-DiHOME mediates thermal hyperalgesia during inflammatory pain. Biochimica et Biophysica Acta (BBA)-Molecular and Cell Biology of Lipids, 1863(7), 669-678.*	73	12.2
53	*Zheng, J., Yang, J., Yang, X., Cao, Z., Cai, S., Wang, B., & Xu, F. (2022). Transcriptome and miRNA sequencing analyses reveal the regulatory mechanism of α-linolenic acid biosynthesis in Paeonia rockii. Food Research International, 155, 111094.*	9	4.5
54	*Wu, Y., Yuan, W., Han, X., Hu, J., Yin, L., & Lv, Z. (2020). Integrated analysis of fatty acid, sterol and tocopherol components of seed oils obtained from four varieties of industrial and environmental protection crops. Industrial Crops and Products, 154, 112655.*	38	9.5
55	*Huang, Z., Yuan, Y., Tan, Z., Zheng, J., Zhang, W., Huang, S., & Li, H. (2023). Metabolomics in combination with network pharmacology reveals the potential anti-neuroinflammatory mechanism of essential oils from four Curcuma species. Industrial Crops and Products, 195, 116411.*	5	5
56	*Gao, H., Yan, P., Zhang, S., Nie, S., Huang, F., Han, H., & Liu, L. (2016). Chronic alpha-linolenic acid treatment alleviates age-associated neuropathology: roles of PERK/eIF2α signaling pathway. Brain, Behavior, and Immunity, 57, 314-325.*	33	4.1
57	*Shams, R., Azizi, A., Hamzei, J., Noroozisharaf, A., Moghadam, S., & Kordrostami, M. (2020). Genetic structure and diversity of Iranian Cannabis populations based on phytochemical, agro-morphological and molecular markers. Industrial Crops and Products, 158, 112950.*	18	4.5
58	*García-Viñuales, S., Sciacca, M. F., Lanza, V., Santoro, A. M., Grasso, G., Tundo, G. R., & Milardi, D. (2021). The interplay between lipid and Aβ amyloid homeostasis in Alzheimer's Disease: risk factors and therapeutic opportunities. Chemistry and Physics of Lipids, 236, 105072.*	24	8
59	*Reemst, K., Lopizzo, N., Abbink, M. R., Engelenburg, H. J., Cattaneo, A., & Korosi, A. (2024). Molecular underpinnings of programming by early-life stress and the protective effects of early dietary ω6/ω3 ratio, basally and in response to LPS: Integrated mRNA-miRNAs approach. Brain, behavior, and immunity, 117, 283-297.*	2	-
60	*Awasthi, S., Kaushik, N., Plaha, N. S., Kaur, V., & Kumar, A. (2024). Exploring lipid health indices and protein quality in ninety Indian linseed varieties by comprehensive analysis of fatty acid composition, lignan content, and amino acid composition. Industrial Crops and Products, 212, 118366.*	5	-
61	*Huang, C., Li, Y., Wang, K., Xi, J., Xu, Y., Si, X., & Huang, J. (2022). Analysis of lipidomics profile of Carya cathayensis nuts and lipid dynamic changes during embryonic development. Food Chemistry, 370, 130975.*	43	21.5
62	*Rodrigues, P. B., Dátilo, M. N., Sant'Ana, M. R., da Silva Nogueira, G. A., Marin, R. M., Nakandakari, S. C. B. R., ... & Cintra, D. E. (2023). The early impact of diets enriched with saturated and unsaturated fatty acids on intestinal inflammation and tight junctions. The Journal of Nutritional Biochemistry, 119, 109410.*	4	4
63	*Alberghini, B., Zanetti, F., Corso, M., Boutet, S., Lepiniec, L., Vecchi, A., & Monti, A. (2022). Camelina [Camelina sativa (L.) Crantz] seeds as a multi-purpose feedstock for bio-based applications. Industrial Crops and Products, 182, 114944.*	16	8
64	*Moura-Assis, A., Afonso, M. S., de Oliveira, V., Morari, J., Dos Santos, G. A., Koike, M., & Cintra, D. E. C. (2018). Flaxseed oil rich in omega-3 protects aorta against inflammation and endoplasmic reticulum stress partially mediated by GPR120 receptor in obese, diabetic and dyslipidemic mice models. The Journal of nutritional biochemistry, 53, 9-19.*	42	7

65	*Yeung, A. W. K., Mocan, A., & Atanasov, A. G. (2018). Let food be thy medicine and medicine be thy food: A bibliometric analysis of the most cited papers focusing on nutraceuticals and functional foods. Food Chemistry, 269, 455-465.*	124	20.7
66	*Fan, R., Toney, A. M., Jang, Y., Ro, S. H., & Chung, S. (2018). Maternal n-3 PUFA supplementation promotes fetal brown adipose tissue development through epigenetic modifications in C57BL/6 mice. Biochimica et Biophysica Acta (BBA)-Molecular and Cell Biology of Lipids, 1863(12), 1488-1497.*	44	7.3
67	*Al-Khudairy, L., Hartley, L., Clar, C., Flowers, N., Hooper, L., & Rees, K. (2015). Omega 6 fatty acids for the primary prevention of cardiovascular disease. Cochrane Database of Systematic Reviews, (11).*	88	9.8
68	*Azrad, M., Turgeon, C., & Demark-Wahnefried, W. (2013). Current evidence linking polyunsaturated fatty acids with cancer risk and progression. Frontiers in oncology, 3, 224.*	224	20.4
69	*Montecillo-Aguado, M., Tirado-Rodriguez, B., Tong, Z., Vega, O. M., Morales-Martínez, M., Abkenari, S., & Huerta-Yepez, S. (2020). Importance of the Role of ω-3 and ω-6 Polyunsaturated Fatty Acids in the Progression of Brain Cancer. Brain sciences, 10(6), 381.*	32	8
70	*Mora, I., Arola, L., Caimari, A., Escoté, X., & Puiggròs, F. (2022). Structured long-chain omega-3 fatty acids for improvement of cognitive function during aging. International journal of molecular sciences, 23(7), 3472.*	26	13
71	*Hooper, L., Al-Khudairy, L., Abdelhamid, A. S., Rees, K., Brainard, J. S., Brown, T. J., & Deane, K. H. (2018). Omega-6 fats for the primary and secondary prevention of cardiovascular disease. Cochrane Database of Systematic Reviews, (7).*	200	33.3
72	*Alagawany, M., Elnesr, S. S., Farag, M. R., El-Sabrout, K., Alqaisi, O., Dawood, M. A., & Abdelnour, S. A. (2022). Nutritional significance and health benefits of omega-3,-6 and-9 fatty acids in animals. Animal Biotechnology, 33(7), 1678-1690.*	58	29

Supplemetal Table S2. The list of countries/territories and journals publishing the 73 reviewed papers (2014-2024).

S/N	Country	Journal	Impact factor
1	Argentina	*Phyton-International Journal of Experimental Botany*	1.3
2	Ireland	*Chemistry and Physics of Lipids*	3.4
3	France	*plant physiology and biochemistry*	6.1
4	Germany	*Phytomedicine*	6.7
		Plant Cell Reports	5.3
5	United kingdom	*International Journal of Food Sciences and Nutrition*	3.5
		BMJ open	2.4
		EFSA Journal	3.3
		International Journal of Food Sciences and Nutrition	3.5
		BMJ open	2.4
		BMC genomics	3.5
		BMC plant biology	4.3
		Journal of Asian Natural Products Research	1.3
		BMC (Springer nature)	2.9
		Plant science	4.2
		Plant biotechnology Journal	11.2
		Journal of Experimental Botany	5.6
		New phytologist	8.3
		Pharmaceutical Biology	3.9
		Plant and cell physiology	3.5
		Food chemistry	8.5
		Food Research International	7.0
		Animal Biotechnology	1.7
		EFSA Journal	3.3
6	Switzerland	*Nutrients*	4.8

		Nutrients	4.8
		Life Sciences	4.1
		International journal of molecular science	4.9
		Genes	2.8
		Plants	4.0
		Frontiers in plant science	4.1
		Frontiers in Oncology	3.5
		Brain Sciences	2.7
		Dementia and Geriatric Cognitive Disorders	2.2
		International Journal of Molecular Sciences	4.9
		Phytochemistry reviews	7.3
7	Netherlands	*Phytochemistry Letters*	1.3
		Industrial Crops and Products	5.6
		Molecular and Cell Biology of Lipids	3.9
		Plant foods for human nutrition	3.1
8	South Korea	***The Plant Journal***	6.2
		ocular surface	5.9
		Metabolic Engineering	6.8
		Agronomy	3.3
		Phytochemistry	3.2
		current Atherosclerosis Reports	5.9
9	United states	*Phytochemistry*	3.2
		Molecular Plant	17.1
		Plant Signaling & Behavior	2.8
		Tropical plant biology	1.8
		Brain, Behavior, and Immunity	8.8
		The Journal of Nutritional Biochemistry	4.8
		Food Reviews International	5.3
